# Border-associated macrophages promote cerebral amyloid angiopathy and cognitive impairment through vascular oxidative stress

**DOI:** 10.21203/rs.3.rs-2719812/v1

**Published:** 2023-04-28

**Authors:** Ken Uekawa, Yorito Hattori, Sung Ji Ahn, James Seo, Nicole Casey, Antoine Anfray, Ping Zhou, Wenjie Luo, Josef Anrather, Laibaik Park, Costantino Iadecola

**Affiliations:** Weill Cornell Medicine

**Keywords:** Border-associated macrophages, CD36, Aβ clearance, neurovascular unit dysfunction, vascular oxidative stress, ARIA

## Abstract

**Background:**

Cerebral amyloid angiopathy (CAA) is a devastating condition common in patients with Alzheimer’s disease but also observed in the general population. Vascular oxidative stress and neurovascular dysfunction have been implicated in CAA but the cellular source of reactive oxygen species (ROS) and related signaling mechanisms remain unclear. We tested the hypothesis that brain border-associated macrophages (BAM), yolk sac-derived myeloid cells closely apposed to parenchymal and leptomeningeal blood vessels, are the source of radicals through the Aβ-binding innate immunity receptor CD36, leading to neurovascular dysfunction, CAA, and cognitive impairment.

**Methods:**

Tg2576 mice and WT littermates were transplanted with CD36^−/−^ or CD36^+/+^ bone marrow at 12-month of age and tested at 15 months. This approach enables the repopulation of perivascular and leptomeningeal compartments with CD36^−/−^ BAM. Neurovascular function was tested in anesthetized mice equipped with a cranial window in which cerebral blood flow was monitored by laser-Doppler flowmetry. Amyloid pathology and cognitive function were also examined.

**Results:**

The increase in blood flow evoked by whisker stimulation (functional hyperemia) or by endothelial and smooth muscle vasoactivity was markedly attenuated in WT^®^Tg2576 chimeras but was fully restored in CD36^−/−^^®^Tg2576 chimeras, in which BAM ROS production was suppressed. CAA-associated Aβ_1–40_, but not Aβ_1–42_, was reduced in CD36^−/−^^®^Tg2576 chimeras. Similarly, CAA, but not parenchymal plaques, was reduced in CD36^−/−^^®^Tg2576 chimeras. These beneficial vascular effects were associated with cognitive improvement. Finally, CD36^−/−^ mice were able to more efficiently clear exogenous Aβ_1–40_ injected into the neocortex or the striatum.

**Conclusions:**

CD36 deletion in BAM suppresses ROS production and rescues the neurovascular dysfunction and damage induced by Aβ. CD36 deletion in BAM also reduced brain Aβ_1–40_ and ameliorated CAA without affecting parenchyma plaques. Lack of CD36 enhanced the vascular clearance of exogenous Aβ. Restoration of neurovascular function and attenuation of CAA resulted in a near complete rescue of cognitive function. Collectively, these data implicate CNS BAM in the pathogenesis of CAA and raise the possibility that targeting BAM CD36 is beneficial in CAA and other conditions associated with vascular Aβ deposition and damage.

## Background

Deposition of amyloid-b (Ab) in cerebral blood vessels (cerebral amyloid angiopathy; CAA) is a key pathological feature of Alzheimer’s disease (AD) [1]. CAA also occurs independently of AD and can either be sporadic or caused by mutations of the amyloid precursor protein (APP) [2]. Amyloid deposition is observed first in the wall of cerebral arterioles wherein the short form of Aβ (Aβ_1−40_) is the prevalent peptide that accumulates [3–5]. CAA has devastating consequences for brain health leading to parenchymal hemorrhages, microbleeds, white matter lesions, and superficial cortical siderosis, and, to date, remains untreatable [2].

Increasing evidence implicates failure of vascular Aβ clearance in the mechanisms of CAA [2, 6]. Ab is released in the extracellular space during neural activity and is promptly removed by various mechanisms, including vascular clearance [6]. Once in the perivascular space, Aβ is transported across the vessel wall into the blood [7–9] or conveyed from the perivascular space to the subarachnoid space where it reaches the CSF [10–13]. From the subarachnoid space Aβ is transported out of the brain via CSF clearance pathways through venous and lymphatic vessels [11, 14].

Cerebrovascular health is essential for vascular clearance of Aβ and alterations in neurovascular function have been implicated in CAA [2]. Suppression of the ability of neural activity to increase cerebral blood flow (CBF) (functional hyperemia), a fundamental homeostatic response of the cerebral microcirculation [15], occurs early in patients with CAA [16–18], whereas Aβ-induced suppression of functional hyperemia and dysfunction of the endothelial regulation of CBF have been linked to CAA in mouse models [13, 19–21]. Aβ-induced vascular oxidative stress and the attendant neurovascular dysregulation and damage have emerged as key factors in CAA both in mouse models [19, 20] and in humans [22]. However, the cellular sources of the reactive oxygen species (ROS) and the related signaling mechanisms remain to be established.

Brain border-associated macrophages (BAM), like microglia, are yolk sack-derived myeloid cells that reach the brain and populate meninges, perivascular spaces, and the choroid plexus [23]. In particular, BAM, leptomeningeal and perivascular macrophages, are closely associated with cerebral blood vessels and a major source of vascular oxidative stress through the ROS-producing enzyme Nox2 [24–26]. CD36, an Aβ-binding scavenger receptor of which gene variants are linked to AD [27, 28], is enriched in BAM and underlies the oxidative stress and neurovascular dysfunction induced by Aβ [29, 30]. In pre-symptomatic (age 3 months) transgenic mice expressing the Swedish mutation of APP (Tg2576), Aβ triggers vascular oxidative stress by activating CD36 in BAM, which, in turn, mediates the attendant suppression of functional hyperemia and endothelial vasoactive function [25]. However, it remains to be established if BAM are responsible for the vascular accumulation of Aβ in older Tg2576 with florid amyloid pathology and cognitive impairment. Therefore, in this study we used a bone marrow (BM) chimera-based approach to investigate the role of BAM in the neurovascular dysfunction, cognitive impairment, vascular pathology and CAA in 15-month-old Tg2576 mice.

## Materials and methods

### Mice

The Institutional Animal Care and Use Committee of Weill Cornell Medicine approved all experimental procedures. Experiments were performed in 12–15 month-old transgenic mice overexpressing the Swedish mutation of the amyloid precursor protein (APP) (Tg2576)[31] or age-matched WT littermates, referred to as WT mice. In bone marrow (BM) chimera experiments, WT and CD36^−/−^ mice were used as BM donors. Some studies used GFP^+^ mice (JAX Stock #006567) as BM donors. All mice were males and derived from in-house colonies [25, 29, 30, 32, 33].

### Bone marrow transplantation

Procedures for BM transplantation have been previously described [24, 25, 34] and are only summarized. Whole-body irradiation was performed in 12-month-old mice (Nordion Gammacell 40 Exactor). Eighteen hours later, mice were transplanted with BM cells (2×10^6^, i.v.) isolated from CD36^−/−^ and WT controls. Mice were housed in cages with sulfamethoxazole (0.12%; w/v) and trimethoprim (0.024%) added to drinking water for the first two weeks. Reconstitution of BM cells was verified five weeks after irradiation by testing the positive CD36 genomic DNA percentage in isolated blood leukocytes [34]. Reference primers sequences were as follows: m_ICAM1_prom.3, 5′-GGACTCACCTGCTGGTCTCT-3′ and m_ICAM1_prom.4, 5′-GAACGAGGGCTTCGGTATTT-3′; target primers sequences were as follows: CD36_1, 5’- −3’ and CD36_2, 5’- −3’, m_Cybb_gt_1, 5’-CTGCTCACCAGCCTCTCTCTA-3’ and m_Cybb_gt_2, 5’-CTGGAACCCCTGAGAAAGGAG-3’ (Invitrogen). qRT-PCR was conducted with 20 ng of DNA, in duplicate 15 µl reactions using the Maxima SYBR Green/ROX qPCR Master Mix (2×) (Thermo Scientific). Chimerism was > 95% for CD36^−/−^ BM chimeras. A PCR cycling protocol consisting of 15 s at 95°C and 1 min at 60°C for 45 cycles was used for quantification CD36 relative expression levels were calculated by the 2 (−ΔΔ CT) method. To study BAM number and distribution after BM transplant in Tg2576 mice, BM from mice expressing GFP (GFP BM) was transplanted into Tg2576 mice or WT littermates at 3 or 12 months of age and the brain distribution of GFP expressing cells was examined at 15 months of age. In some experiments, GFP BM was transplanted into irradiated WT mice with head shielding at 3 months and GFP-expressing cells were examined 3 months later.

### CBF measurement

#### Surgical procedures:

As described in detail elsewhere [29, 30, 32, 33], mice were anesthetized with isoflurane (induction, 5%; surgery, 1.5%) and maintained with urethane (750 mg/kg; i.p.) and α-chloralose (50 mg/kg; i.p.). A femoral artery was cannulated to record arterial pressure and collect blood samples for blood gas analysis. The trachea was intubated and mice were artificially ventilated with a mixture of N_2_ and O_2_. Arterial blood pressure (80–90 mmHg), blood gases (pO_2_, 120–140 mmHg; pCO_2_, 30–40 mmHg; pH, 7.3–7.4), and rectal temperature (37°C) were monitored and controlled. Throughout the experiment, the level of anesthesia was monitored by testing motor responses to tail pinch. The somatosensory cortex was exposed through a small craniotomy (2×2 mm). The dura was removed, and the exposed cortex was continuously bathed with a modified Ringer’s solution (36–37°C; pH: 7.3–7.4)(see ref. [35] for composition). CBF was continuously monitored at the site of superfusion with a laser-Doppler probe (Vasamedic, St. Paul, MN) positioned stereotaxically on the neocortical surface and connected to a computerized data acquisition system. CBF values were expressed as a percent increase relative to the resting level. Resting CBF is reported as arbitrary laser-Doppler perfusion units (LDU). Zero values for CBF were obtained after stopping the heart at the end of the experiment.

##### Experimental protocol

CBF recordings were started after arterial pressure and blood gases were stable. To test functional hyperemia, the CBF response evoked by gently stroking the whiskers with a cotton-tipped applicator for 60 sec was recorded. To test endothelium-dependent vasodilatation, acetylcholine (10 µM, Sigma), the Ca^2+^ ionophore A23187 (3 µM; Sigma) or bradykinin (50 µM; Sigma) was topically superfused for 3–5 min and the evoked CBF increases recorded. To test smooth muscle function, the CBF responses to adenosine (400 µM, Sigma) or to the NO donor S-Nitroso-N-acetyl-DL-penicillamine (SNAP; 50 µM, Sigma) were examined [25, 30, 36]. All pharmacological agents were dissolved in a modified Ringer’s solution [35]. The increase in CBF produced by hypercapnia was tested by introducing 5% CO_2_ in the ventilator to increase arterial pCO_2_ up to 50–60 mmHg. Once a stable increase in CBF was obtained for 3–5 min, pCO_2_ was returned to normocapnia.

### Intracerebroventricular injection of dextran

BAM were identified by their ability to phagocytize dextran [24, 25, 37]. For dextran injections, 10 µl of Alexa Fluor→ 680 dextran (10,000 MW, anionic, fixable, ThermoFisher Scientific, D34680; 2.5 mg/ml) in saline or saline alone were slowly injected into the cerebral ventricles with a glass micropipette through a burr hole drilled on the right parietal bone [25]. BAM labeling was examined 24 hrs later.

### Labeling cortical blood vessels with DiO

Cortical blood vessels were labeled with the lipophilic dye DiO [DiOC18(3) (3,3’-Dioctadecyloxacarbocyanine Perchlorate)], as described [25, 38]. Briefly, mice were anesthetized (5% isoflurane) and transcardially perfused with PBS (2 ml) followed by DiO (1:50, V-22886, Molecular Probes; 5ml/mouse) and then by 4% paraformaldehyde (PFA). Brains were harvested and post-fixed in 4% PFA overnight, then cut (thickness 150 µm) using a vibratome and examined under the confocal microscope (Leica SP8).

### Immunohistochemistry

Mice were anesthetized with sodium pentobarbital (120 mg/kg, i.p.) and perfused transcardially with PBS followed by 4% PFA in PBS. Brains were removed, post-fixed overnight, and sectioned coronally in a vibratome (section thickness: 40 µm). In some experiments, cortices were dissected out, flattened and post-fixed overnight. The cortices were tangentially sectioned as above. Free-floating brain sections were permeabilized with 0.5% Triton X-100 and non-specific binding was blocked with 1% of normal donkey serum. Sections were randomly selected and incubated with the primary antibodies CD206 (clone MR5D3, rat polyclonal, 1:200, Serotec), CD36 (mouse monoclonal, 1:500, BD Biosciences), Glut-1 (rabbit polyclonal, 1:200, Calbiochem), Iba-1 (rabbit polyclonal, 1:500, Wako Chemicals), a-Actin (rabbit polyclonal, 1:300, abcam), or GFAP (mouse monoclonal, 1:1000, Sigma) overnight at 4°C. After washing, brain sections were incubated with a Cy5- or a FITC-conjugated secondary antibody (1:200; Jackson ImmunoResearch Laboratories), mounted on slides and imaged with a confocal microscope (Leica SP8). In some experiments, brain sections were con-stained with thioflavin-S (0.5%) to assess amyloid plaques and CAA. The specificity of the immunofluorescence was verified by the omission of the primary and/or secondary antibody or blocking the antigen. All quantifications were performed by investigators blinded to the treatment on randomly selected fields within the somatosensory cortex.

### Identification and quantification of BAM in the somatosensory cortex

BAM were identified by well-established criteria, including expression of CD206, ability to phagocytize dextran and perivascular location [24, 25, 39, 40]. The association with cortical blood vessels was confirmed by co-labeling with the endothelial marker Glut-1 (rabbit polyclonal, 1:200, Calbiochem), a-Actin (rabbit polyclonal, 1:300, abcam), or DiO [25, 38]. For CD206^+^ BAM, randomly selected fields (20x objective; 4 confocal images/mouse; n = 5 mice/group) within the somatosensory cortex were analyzed. For dextran^+^ BAM, a representative coronal section from each mouse was reconstructed from tiled images taken with the confocal microscope, and the whole somatosensory cortex (n = 5/group) was analyzed. ImageJ (NIH) was used for all image analyses.

### ROS measurement

ROS production was assessed in vivo by dihydroethidine (DHE) microfluorography [24, 25, 29, 41, 42]. BM-transplanted WT or Tg2576 mice were first injected with icv dextran (see above). The day after the dextran injection, DHE (10 mg/kg; Invitrogen) was infused into the jugular vein in mice under isoflurane anesthesia. Sixty minutes after DHE administration, mice were transcardially perfused with DiO to label cerebral blood vessels as described above and before [25, 38]. Coronal brain sections were then cut through the cortex underlying the cranial window, and ROS-dependent fluorescence associated with BAM was quantified as described previously [24, 25, 41].

### Brain Aβ measurement

Brain Aβ was measured using an ELISA-based assay as described previously [33]. Briefly, the left hemispheres of the mice used for CBF studies were homogenized with RIPA followed by a 5.5 M guanidine buffer containing a cocktail of protease inhibitors (1:1000; Roche). Aβ measured after the RIPA extraction represented the soluble pool of Aβ, whereas Aβ measured after guanidine extraction represented the insoluble pool. The homogenates were diluted with a cold sample dilution buffer (1% bovine serum albumin in PBS and 0.05% Tween 20 [PBST]) before measurement of Aβ_1–40_ or Aβ_1–42_. Guanidine-solubilized samples were diluted with a cold sample dilution buffer to a final concentration of 0.5 M GuHCl. Samples were loaded onto plates coated with an antibody that specifically recognizes the C-terminal domain of Aβ_1–42_ (21F12) or Aβ_1–40_ (2G3) as the capture antibody, and biotinylated 3D6 was used for detection. The immunoreactivity signal after incubation with horseradish peroxidase-conjugated streptavidin (Research Diagnostics) was developed with a TMB substrate (Thermo Fisher Scientific) and read on a Synergy H1 Hybrid plate reader (BioTek). Levels of Aβ were calculated using a standard curve generated with recombinant human Aβ (American Peptide Company). Levels of Aβ in brain homogenates were determined in triplicate, normalized to protein content, and expressed as the amount of Aβ per milligram of protein. Concentrations in picomoles per milligram of brain tissue were calculated by comparing the sample absorbance with the absorbance of known concentrations of synthetic Aβ_1–40_ and Aβ_1−42_.

### Amyloid burden, CAA and smooth muscle cell fragmentation.

#### In vivo CAA imaging

We imaged pial vessel CAA using 2-photon microscopy. Optical access to the brain was achieved through a polished and reinforced thinned skull preparation sealed with cyanoacrylate glue and a cover glass [26, 32]. Mice were allowed at least two weeks to recover from window implantation. To label Aβ deposits, methoxy-X04 (Tocris, dissolved in DMSO at 100 mM) was intraperitoneally injected one day before imaging at a dose of 1 mg/100 g [43]. To fluorescently label the blood vessel, Texas Red dextran (40 µl, 2.5%, molecular weight (MW) = 70,000 kDa, Thermo Fisher Scientific) in saline was injected retro-orbitally immediately before imaging. Imaging was performed on a commercial 2-photon microscope (FVMPE; Olympus) with XLPlan N 25×1.05 NA objective. Excitation pulses came from a solid-state laser (InSight DS+; Spectraphysics) set at 830 nm wavelength. Image stacks were acquired through Fluoview software. During imaging, anesthesia was maintained with ~ 1.5% isoflurane in an oxygen/nitrogen mix (21% oxygen), with slight adjustments made to the isoflurane to maintain the respiratory rate at ~ 1 Hz. Animals were kept at 37°C with a feedback-controlled heating pad. Two photon images are average projection of three-dimensional stacks using ImageJ (NIH).

##### Amyloid burden:

Tangential brain sections were first incubated with α-actin (1:300, rabbit polyclonal; Abcam) or Glut-1 (rabbit polyclonal, 1:200, Calbiochem) antibody for 48 h, and, after washing, followed by Alexa 647-conjugated anti-rabbit IgG (1:200, Jackson ImmunoResearch). After mounting and drying on slides, brain sections were rehydrated with PBS and refixed with 4% PFA for 10 min. After washing, sections were labeled with 0.5% (wt/vol) thioflavin-S in 50% (vol/vol) ethanol for 10 min to identify CAA. Confocal images were obtained with an Alexa 488 filter for thioflavin-S and an Alexa 647 filter for α-actin or Glut-1. Images of α-actin or Glut-1 with thioflavin-S were acquired, and the number of α-actin^+^ or Glut-1^+^ was quantified. The CAA burden was expressed by the number of neocortical parenchymal vessels positive for both thioflavin-S and α-actin or Glut1 [20, 30].

##### Smooth muscle cell fragmentation:

To quantify Aβ-associated fragmentation of smooth muscle cells in pial vessels [20, 30], brain sections were incubated with anti-Aβ (4G8, 1:1,000, mouse; Covance) and the smooth muscle marker anti-α-actin (1:300, rabbit, Abcam) for 48 hr. After washing, sections were labeled with Alexa 488-conjugated anti-rabbit IgG (1:200; Jackson ImmunoResearch) and Alexa 647-conjugated anti-rabbit IgG (1:200; Jackson ImmunoResearch). Pial arterioles (n = 30–50/group) positive for Aβ and α-actin, ranging in diameter from 20 to 100 µm, were randomly imaged by confocal microscope (63x). The fragmentation of smooth muscles was quantified by counting the number of α-actin fragments of each arteriole using ImageJ, expressed as the fragmentation index: 100 − [(1/number of α-actin fragments) x 100].

#### Brain Aβ clearance

##### Aβ measurement in brain and plasma:

CD36^−/−^ and WT mice were anesthetized with 1.5% isoflurane and placed on a stereotaxic device. A burr hole was drilled into the somatosensory cortex at coordinates: −1.58 mm anterior to bregma, 2.5 mm lateral, and 0.4 mm from the dura. Human Aβ_1−40_ (100 mmol/L; rPeptide, Watkinsville, GA) was slowly injected in a volume of 1 µl using an Ultramicropump (World Precision Instruments, Sarasota, FL). One hour later blood samples were collected from the superior sagittal sinus (SSS) and heart, and the site of neocortical injection was sampled. Samples were stored at −80 °C until assay. Plasma and brain Aβ_1−40_ levels were quantified using V-PLEX Aβ Peptide Panel 1 (4G8) [Stock # K15199E-2, Meso Scale Discovery (MSD), Rockville, MD, USA] according to the manufacturer instructions.

#### Neocortex

For determination of Aβ neocortical clearance, after surgical preparation of CD36^−/−^ and WT mice, Cy5-conjugated Aβ_1−40_ (100 mmol/L) was slowly injected into the neocortex at the same coordinates as above in a volume of 1 µl using an Ultramicropump (World Precision Instruments, Sarasota, FL). One hour later, brains were removed and sectioned with a vibratome (40 µm thickness) and imaged with a confocal microscope The intensity profiles of Cy5-conjugated Aβ_1−40_ were quantified with ImageJ (NIH).

##### Striatum:

For determination of Aβ clearance in the striatum [8], a guide cannula was placed in the left striatum of CD36^−/−^ and WT mice at coordinates: −0.10 mm anterior to bregma, 2.2 mm lateral, and 2.8 mm from the dura. Mice were then allowed to recover for 4–6 hours after surgery [8, 44]. Then, Cy5-conjugated Aβ_1−40_ (1 µmol/L) was slowly injected in a volume of 0.5 µl using an injection needle. FITC labelled inulin (1 µmol/L), an inert reference molecule neither actively transported across the BBB nor retained within the brain [8, 45], was co-injected in the same mice. Thirty minutes after the co-injection, brains were sectioned in a cryostat (thickness 20 µm), and images were acquired with a confocal microscope. The intensity profiles of Aβ_1−40_ and Inulin were quantified with ImageJ.

### Cognitive testing

Methods for cognitive testing have been described in detail previously [32, 46, 47] and are only summarized.

#### Barnes maze

Mice were studied in groups of 10 with the inter-trial interval (20–30 minutes). All the mice examined were trained with an escape hole located in the same location across trials. No habituation trial was performed. The acquisition phase consisted of 4 consecutive training days with four trials per day. After each trial, mice remained in the escape box for 60 seconds before being returned to their home cages. Mice were allowed 3 minutes for each trial to locate the escape hole. Probe trials were performed on day 5, 24 hr after the last acquisition test. After removing the escape hole, mice were placed in a start quadrant of the Barnes maze and allowed to explore for 90 seconds. Then, we analyzed (a) latency to enter the escape hole during the acquisition phase (escape latency) and (b) time spent in the escape quadrant in the probe trial.

##### Nesting test:

In the evening mice were placed in individual cages with pre-weighted nestlets (3g/cage) and the next morning the remaining nestlets not assembled into a nest were weighed [48]. The nests were scored on a 5-point rating scale based on the remaining nestlet ratio and shredded conditions: 1, nestlet not noticeably torn (> 90% nestlet untorn); 2, nestlet partially torn (50–90% nestlet untorn); 3, nestlet mostly shredded but not recognizable nest built (< 50% nestlet untorn); 4, nest built recognizable but flat (< 10% nestlet untorn); 5, nest near perfectly built like a crater (< 10% nestlet untorn) with walls higher than mouse body height for more than 50% of its circumference. Shredded nestlets were expressed as %.

#### Statistics

##### Data analyses.

Sample sizes were determined by power analysis using G*Power (v.3.1.9.2) based on previous works published by our lab on CBF regulation cognitive testing [25, 32]. The experiments were randomized based on the random number generator (https://www.random.org) and were performed and analyzed in a blinded fashion whenever possible. Data and image analyses were done using ImageJ 1.54c (NIH) or Prism 9 for MacOS (GraphPad Software). Data were tested for normal distribution by the D’Agostino–Person test and for outliers by the Grubbs’ test (extreme studentized deviate). Two-group comparisons were analyzed using paired or unpaired two-tailed t-test, as indicated. Multiple comparisons were evaluated by one-way or two-way analysis of variance (ANOVA) and Tukey’s test. Differences were considered statistically significant for probability values less than 0.05. Data are expressed as the mean ± S.E.M.

## Results

### 1. Bone marrow transplantation at 12 months of age replaces leptomeningeal and perivascular BAM and not microglia

**Figure 1:**
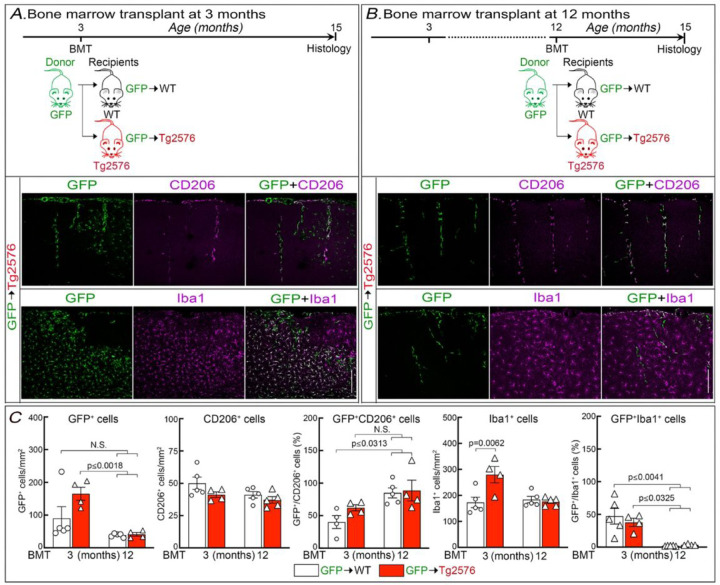
Outcome of GFP^+^ BM transplantation at 3 or 12 months of age followed by identification of GFP^+^ cells at 15 months of age. ***A:***GFP^+^ BM was transplanted into Tg2576 mice or WT littermates at 3 months and GFP^+^ cell identity determined at 15 months of age (See Fig. S1 for WT groups). In Tg2576 mice, GFP^+^ cells were seen surrounding cerebral blood vessels, which were also positive for the BAM marker CD206, as well in the parenchyma, which had the morphology of microglia and were strongly Iba1^+^. ***B:*** GFP^+^ BM was transplanted in Tg2576 mice or WT littermates at 12 months and GFP^+^ cell identity determined at 15 months of age. In Tg2576 mice, GFP^+^/CD206^+^ cells were seen surrounding cerebral blood vessels, no GFP^+^/Iba1^+^ were observed in the brain parenchyma. ***C:*** Number of GFP^+^ and CD206^+^ cells, % GFP^+^CD206^+^ cells, number of Iba1^+^ cells, and % GFP^+^/Iba1^+^ cells at 15 months of age after BM transplant at 3 or 12 months. N=4–5/group; Two-way ANOVA with Tukey’s test; mean±SEM; scale bar in ***A*** and ***B***, 200 µm; data presented as mean±SEM.

To investigate the role of BAM in CAA we used a well-established BM transplantation strategy to replace WT BAM with BAM lacking CD36 in 15-month-old Tg2576 mice with CAA. The time interval after BM transplantation determines whether the myeloid cell engraftment is limited to perivascular space and leptomeninges or involves also parenchymal microglia [24, 25, 49–51]. Therefore, we first established the time lag between BM transplantation and outcome assessment (neurovascular regulation, amyloid pathology and cognition) that restricts engraftment to BAM. To this end, after total body irradiation, we transplanted GFP^+^ BM in 3- or 12-month-old Tg2576 mice and assessed myeloid cell repopulation at 15 months of age. Age-matched littermates served as WT controls. Transplantation of GFP^+^ BM resulted in accumulation of GFP^+^ cells in the brain in both groups of mice. However, mice transplanted at 3 months of age and examined at 15 months exhibited, in addition to GFP^+^ perivascular and leptomeningeal cells expressing the BAM marker CD206, also parenchymal GFP^+^ cells with a microglial phenotype expressing high levels of Iba1 and more abundant in Tg2576 mice (Fig. 1A,C; Fig. S1A). In contrast, when 12-month-old mice were transplanted, GFP^+^ cells expressing the BAM marker CD206 were observed in perivascular and leptomeningeal compartments, while Iba1^+^ cells were GFP negative (Fig. 1B,C; Fig. S1B). No differences in the number of CD206^+^ cells were observed between Tg2576 mice and littermates (Fig. 1C). Head shielding completely prevented the repopulation of the brain by GFP^+^ cells (Fig. S2) and was not used. Based on these observations in subsequent experiments mice were transplanted at 12 months of age and assessed for neurovascular physiology, amyloid pathology, and cognition at 15 months.

### 2. CD36 deletion in BAM prevents neurovascular dysfunction

**Figure 2:**
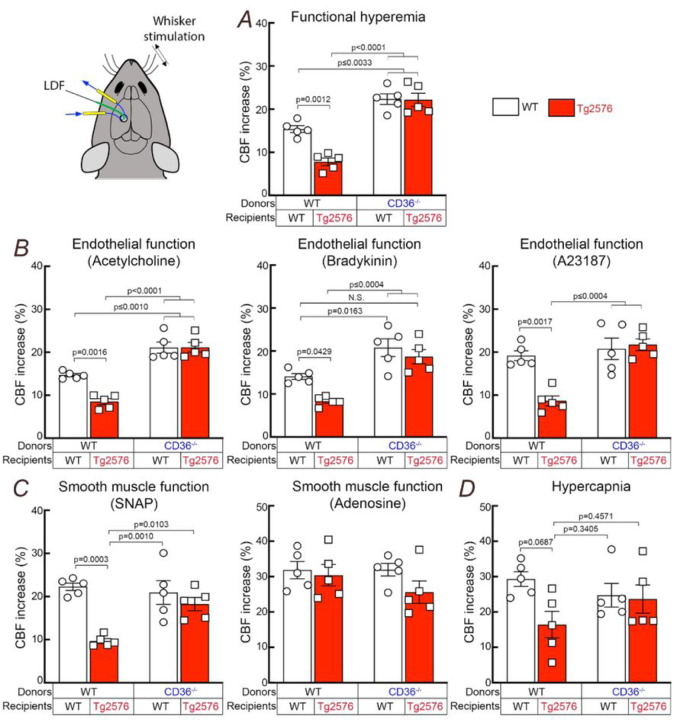
Deletion of CD36 in BAM rescues neurovascular dysfunction in 15-month-old Tg2576 mice. ***A:*** WT^®^Tg2576 chimeras exhibit an attenuated increase in CBF during whisker stimulation (functional hyperemia), which is completely restored in CD36^−/−^^®^Tg2576 chimeras. ***B:*** CBF responses to neocortical application of the mechanistically distinct endothelium-dependent vasodilators acetylcholine, bradykinin, and the Ca^2+^ ionophore A23187 are attenuated in WT^®^Tg2576 chimeras and are restored in CD36^−/−^^®^Tg2576 chimeras. ***C:*** The attenuation of CBF responses to neocortical application the NO donor SNAP in WT^®^Tg2576 is normalized in CD36^−/−^^®^Tg2576 chimeras. CBF responses to adenosine are not attenuated in WT^®^Tg2576 and remain normal in CD36^−/−^^®^Tg2576 chimeras ***D:*** CBF response to the potent vasodilator hypercapnia has a trend toward to attenuation in WT ^®^Tg2576 which was not observed in CD36^−/−^^®^Tg2576 chimeras. N=5/group; two-way ANOVA with Tukey’s test; data presented as mean±SEM.

We then proceeded to determine if CD36 deletion in BAM ameliorates the neurovascular dysfunction in 15 months old Tg2576 mice. First, we verified that CD36 was deleted from BAM in Tg2675 mice transplanted with CD36^−/−^ BM. To this end, we studied BAM CD36 expression in WT and Tg2576 mice transplanted with CD36^+/+^ (WT→WT; WT→Tg2576) or CD36^−/−^ BM (CD36^−/−^→WT; CD36^−/−^→Tg2576). In WT→WT chimeras, CD36 immunoreactivity was observed in perivascular cells expressing the BAM marker CD206 (Fig. S3A,B), and was increased in WT→Tg2676 chimeras (Fig. S3A,B,E), consistent with the previously reported vascular CD36 upregulation in Tg2576 mice [29]. CD36 immunoreactivity in BAM was not observed in mice receiving CD36^−/−^ BM (Fig. S3), attesting to efficient CD36 deletion in BAM. We then used laser-Doppler flowmetry to examine cerebrovascular function in 15 month-old BM chimeras anesthetized and equipped with a cranial window [29, 32, 36, 47]. First, we tested functional hyperemia, a response profoundly attenuated in Tg2576 mice [52]. As anticipated, functional hyperemia induced by mechanical stimulation of the facial whiskers was suppressed in WT→Tg2676 chimeras, compared to WT→WT (Fig. 2A), but was completely rescued in CD36^−/−^ → Tg2676 chimeras. Interestingly, CD36^−/−^→WT chimeras exhibited improved functional hyperemia implicating BAM CD36 also in the attenuation of functional hyperemia observed in aging [41]. Since the ability of cerebral endothelial cells to regulate CBF is also suppressed in Tg2576 mice [53], we tested if endothelial function was also improved by CD36^−/−^ BM transplantation. To this end, we studied the increase in CBF produced by bathing the exposed cerebral cortex with mechanistically distinct vasoactive agents: the endothelial nitric oxide-dependent vasodilator acetylcholine [54], the vasodilator acting through endothelial prostanoids bradykinin [55], or the receptor independent endothelium-dependent vasodilator, the Ca^2+^ ionophore A23187 [56–58]. We found that the attenuation of these endothelial responses in WT→Tg2676 was completely reversed in CD36^−/−^→Tg2676 chimeras (Fig. 2B). Finally, since CAA leads to damage and loss of SMC [30], we also tested smooth muscle vasoreactivity using the cAMP-dependent smooth muscle relaxant adenosine [59], the cGMP-dependent NO donor SNAP [60, 61], and the potent cerebrovasodilator hypercapnia (PaCO_2_: 50–60mmHg). CBF responses to SNAP and hypercapnia were reduced in WT→Tg2676 but were markedly improved in CD36^−/−^→Tg2676 chimeras (Fig. 2C,D). CBF responses to adenosine were not attenuated in any group (Fig. 2D). These data demonstrate a complete restoration of neurovascular function by BAM CD36 deletion in CD36^−/−^→Tg2676 chimeras.

### 3. CD36 deletion in BAM prevents vascular oxidative stress

**Figure 3:**
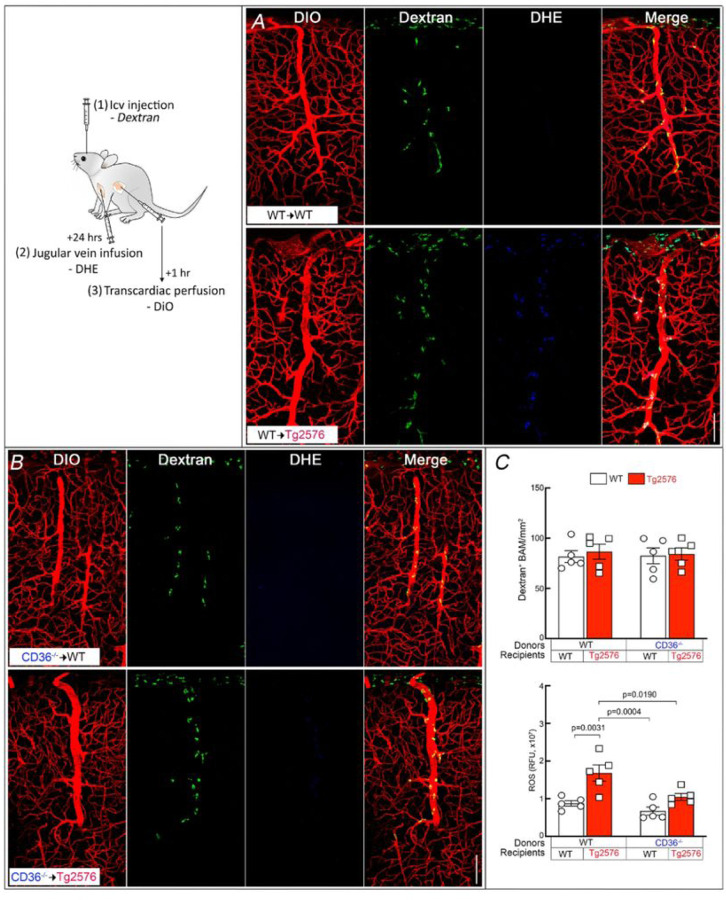
CD36^−/−^ BM transplant in Tg2576 mice suppresses ROS production in BAM. Mice received an icv injection of FITC-dextran to label BAM and, 24 hrs later, the ROS marker DHE was injected into the jugular vein. The vascular marker DiO was injected transcardiacally at the end of the experiment. ***A:*** BAM of WT→Tg2576 mice have more DHE signal than WT→WT. ***B:*** The DHE signal is reduced in CD36^−/−^^®^Tg2576 chimeras. ***C:*** Quantification of the numbers of BAM in the BM chimeras studied showing no differences among groups (top). Quantification of the DHE signal demonstrating reduced signal in CD36^−/−^^®^Tg2576 chimeras (bottom). RFU: relative fluorescent units. N=5/group; two-way ANOVA with Tukey’s test; scale bar, 100 µm; data presented as mean±SEM.

The neurovascular dysfunction induced by Ab is mediated by vascular oxidative stress [41, 62–64]. Therefore, we sought to determine if CD36 deletion in BAM suppresses ROS production in these cells. To this end, BAM were labelled by icv injection of FITC-dextran and, one day later, ROS production was assessed in BAM using DHE as a marker (Fig. Figure 3) [25, 38]. The number of BAM did not differ between groups (Fig. 3A–C), but the increase in BAM ROS production observed in WT→Tg2676 did not occur in CD36^−/−^→Tg2676 chimeras (Fig. 3A–C). These data collectively demonstrate that deletion of CD36 in BAM suppresses ROS production in BAM in 15-month-old Tg2576 mice.

### 4. CD36 deletion in BAM ameliorates CAA without reducing amyloid plaques

**Figure 4:**
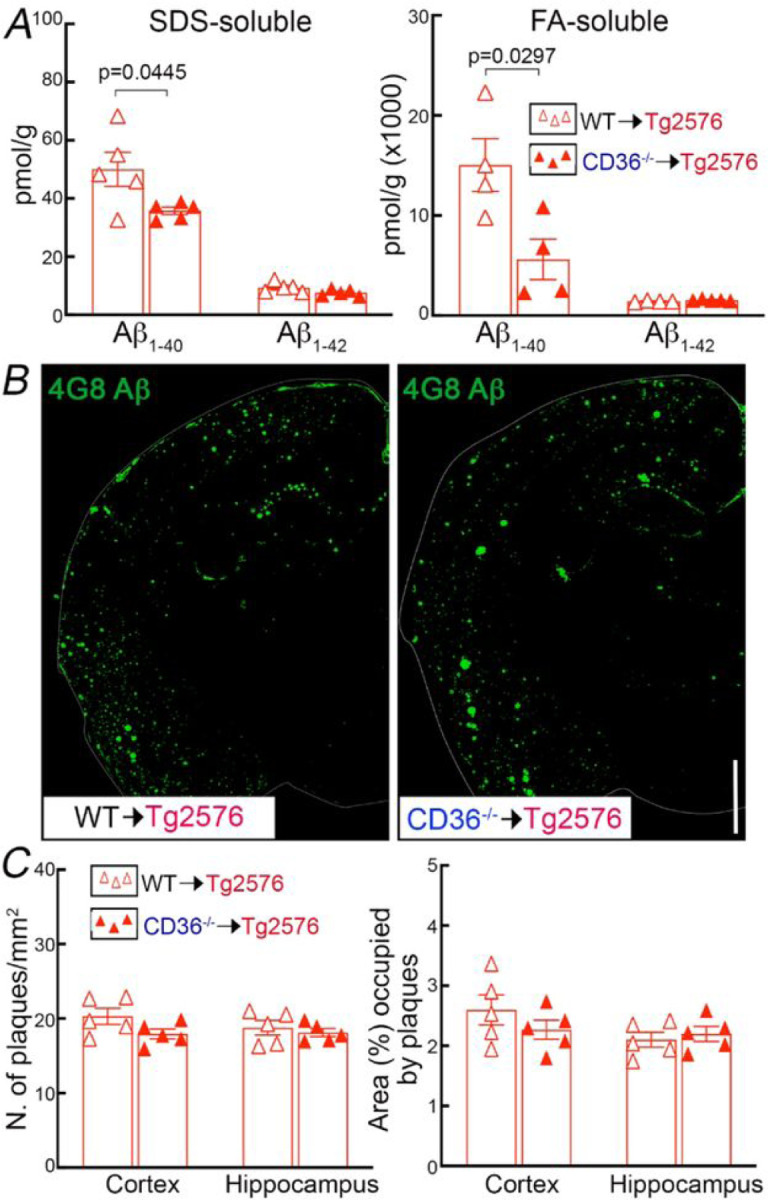
Deletion of CD36 in BAM reduces Aβ_1–40_, but not Aβ_1–42_ or amyloid plaque load, in 15-month-old Tg2576 mice. ***A:*** SDS-soluble and FA-soluble (SDS-insoluble) Aβ_1–40_ are reduced in CD36^−/−^→Tg2576 compared with WT^®^Tg2576 chimeras. ***B:*** Amyloid plaque load assessed by 4G8 immunocytochemistry. ***C:*** Plaque number/mm^2^ and percent of area occupied by plaques do not differ between WT→Tg2576 and CD36^−/−^ →Tg2576. N=5/group; two-way ANOVA with Tukey’s test; data presented as mean±SEM.

**Figure 5:**
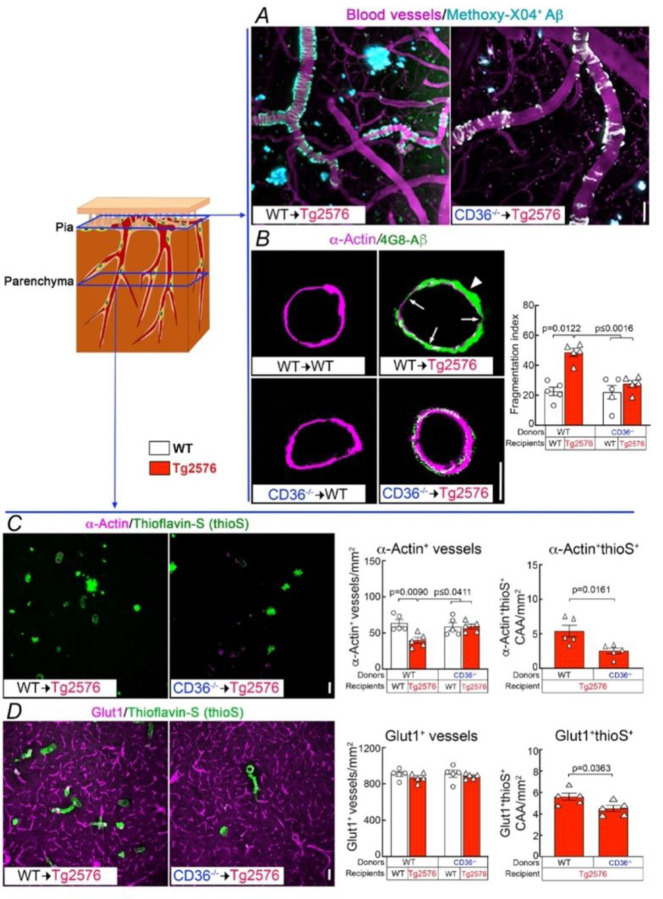
CD36 deletion in BAM attenuates pial and parenchymal CAA in 15-month-old Tg2576 mice. Schematic drawing depicting the location of the pial and parenchymal vessels studied. ***A-B:*** Pial CA ***A:** In vivo* two-photon excited fluorescent images illustrating methoxy-XO4^+^ amyloid deposits (Cyan) around somatosensory cortex blood vessels (magenta) identified by retroorbital injection of 70 kDa Texas Red dextran. Methoxy-XO4^+^ Aβ deposits are reduced in CD36^−/−^→Tg2576 compared to WT→Tg2576 mice. ***B:*** Smooth muscle cells are fragmented (arrows) near dense amyloid deposits (arrowhead) in WT→Tg2576 chimeras compared with WT→WT. The barograph shows quantification of fragmentation (fragmentation index, see methods). ***C-D***: Parenchymal CAA in tangential cortical sections double labelled with the smooth muscle cell marker a-actin or the endothelial marker Glut1 and thioS. a-actin is reduced in WT→Tg2576 but not in CD36^−/−^→Tg2576 chimeras, and the number of a-actin^+^ thioS^+^ vessels is reduced in CD36^−/−^→Tg2576 chimeras (***C***). The number of Glut1^+^ vessels is comparable between groups, but the number of Glut1^+^thioS^+^ vessels is reduced in CD36^−/−^→Tg2576 chimeras (***D***). The barographs show quantification. N=5/group; two-way ANOVA with Tukey’s test; scale bars in A-D, 50 µm. Data presented as mean±SEM.

Next, we examined the impact of CD36 deletion in BAM on brain Ab and on amyloid-b accumulation in brain parenchyma and blood vessels. Brain Ab_1 − 40_ was reduced in CD36^−/−^→Tg2676, compared to WT→Tg2676 mice, while Ab_1 − 42_ was not reduced (Fig. 4A). When examining parenchymal amyloid deposition, we found that amyloid plaques were not reduced (Fig. 4B–C), but CAA was markedly attenuated both in pial and parenchymal microvessels in CD36^−/−^→Tg2676 compared to WT→Tg2676 chimeras (Fig. 5). The CAA reduction was associated with less smooth muscle cell fragmentation and loss (Fig. 5A–C). However, the overall number of vessels assessed by Glut1 immunocytochemistry was comparable between CD36^−/−^→Tg2676 and WT→Tg2676 chimeras (Fig. 5D) suggesting that the smooth muscle cell injury was not due to global vascular degeneration. No differences in the number of CD206^+^, Iba1^+^, or GFAP^+^ cells were observed between mice transplanted with WT or CD36^−/−^ BM (Fig. S4). These observations, collectively, suggest CD36 deletion in BAM may promote the disposal of Aβ_1−40_, the vasotropic form of the peptide, ameliorating vascular but not parenchymal Aβ deposition.

### 5. CD36 deletion in BAM rescues cognitive impairment

**Figure 6:**
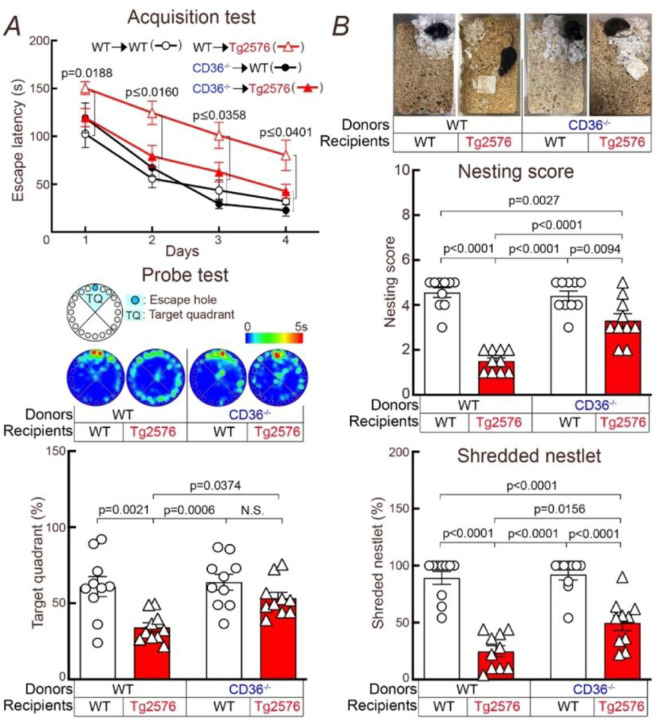
CD36 deletion in BAM prevents cognitive dysfunction in 15-month-old Tg2576 mice. **A:** Escape latency is increased in WT→Tg2576 and normalized in CD36^−/−^→Tg2576 chimeras. Similarly, CD36^−/−^→Tg2576 chimeras identify the target quadrant more reliably at the probe test. **B:** Nest building abilities are improved in CD36^−/−^→Tg2576 chimeras, compared to WT→Tg2576 chimeras, as shown by a significantly higher nesting score and % shredded nestlets. N=10/group; data were analyzed by two-way ANOVA and Tukey’s test, except for the acquisition test which was analyzed by two-way ANOVA with repeated-measures and Tukey’s test. Data presented as mean ± SEM.

Next, we sought to determine if the improvement of neurovascular function and CAA in CD36^−/−^ →Tg2676 chimeras was associated with improvement of cognitive function. We found that WT→Tg2676 chimeras took more time to identify the escape hole at the Barnes maze and had more difficulty identifying the target quadrant once it was removed (probe test) (Fig. 6A). In contrast, CD36^−/−^→Tg2676 chimeras exhibited marked improvements in escape latency and performance at the probe test (Fig. 6A). Nest building capacity was also improved in CD36^−/−^→Tg2676 compared to WT→Tg2676 chimeras (Fig. 6B). Therefore, deletion of CD36^−/−^ in BAM resulted in a marked improvement of cognitive function.

### 6. CD36 depletion promotes brain Aβ clearance

**Figure 7:**
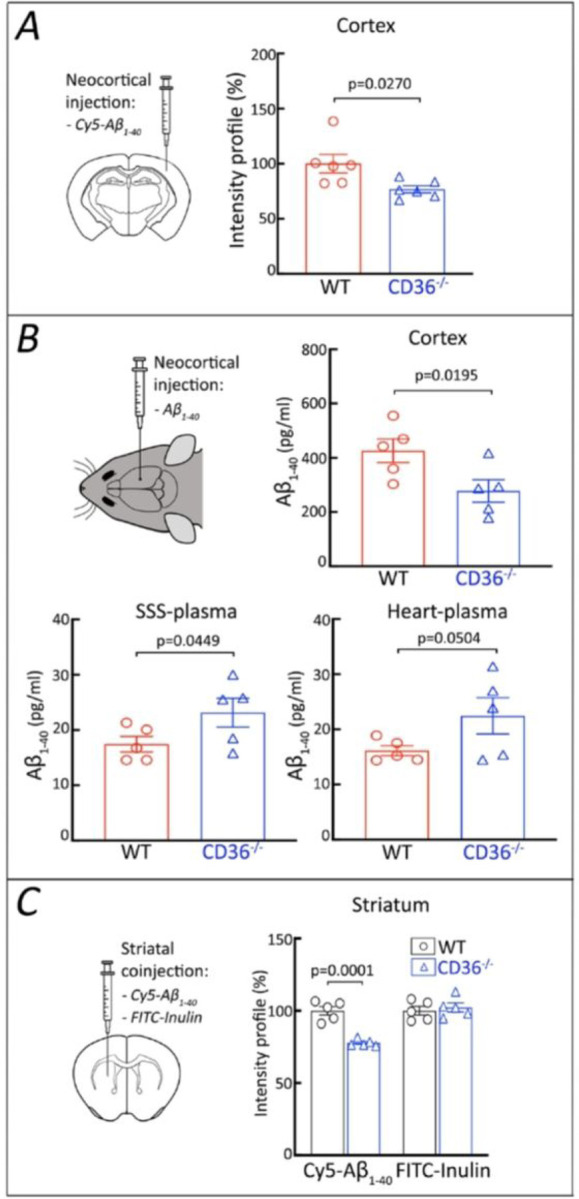
Aβ_1–40_ is cleared more effectively from cortex and striatum in CD36^−/−^ mice than in WT mice. ***A:*** Cy5-conjugated Aβ_1–40_ was injected into the somatosensory cortex of CD36^−/−^ and WT mice. One hour later, the Cy5-Aβ_1–40_ fluorescence at the injection site was lower in CD36^−/−^ than in WT mice. ***B:*** Six-month-old CD36^−/−^ and WT mice were injected with human Aβ_1–40_ into somatosensory cortex. One hour later, Aβ_1–40_ levels at the injection site were lower in CD36−/− mice, while Aβ_1–40_ levels in blood sampled from the superior sagittal sinus (SSS) or the heart were higher, suggesting more efficient Aβ_1–40_ tissue clearance. ***C:*** Cy5-Aβ_1–40_ and FITC-labelled inulin were co-injected into the striatum of CD36^−/−^ and WT mice. Thirty min later, the Cy5-Aβ_1–40_ fluorescence was lower in CD36^−/−^ mice than in WT mice, while the FITC-inulin fluorescence, a reference signal, was not affected. Data were analyzed by the two-tailed unpaired T-test. Data presented as the mean±SEM.

Neurovascular dysfunction impairs the brain elimination of Ab by suppressing its trans-vascular and perivascular clearance [13, 65]. Therefore, the reduction in brain Ab_1 − 40_ and CAA in CD36^−/−^→Tg2676 chimeras raises the possibility that CD36 deficiency promotes Ab_1 − 40_ neurovascular clearance [66] by counteracting the well-described neurovascular dysfunction induced by Ab_1 − 40_ [29]. To gain initial insight into this possibility we injected Cy5-labelled Ab_1 − 40_ into the neocortex of WT and CD36^−/−^ mice and assessed the local clearance of the tracer. We observed that Cy5-Ab_1 − 40_ was cleared more efficiently in CD36^−/−^ than in WT mice (Fig. 7A). Thus, we found that in CD36^−/−^ mice Ab_1 − 40_ levels were lower in the neocortex and higher in superior sagittal sinus blood, the venous effluent from the neocortex, or peripheral blood (Fig. 7B). Similarly, Cy5-Ab_1 − 40_ injected into the striatum was cleared more effectively in CD36^−/−^ mice, while inulin, a reference marker that is neither transported across the BBB nor retained by the brain [45], was cleared equally well in WT and CD36^−/−^ mice (Fig. 7C). Therefore, these initial studies suggest that Aβ_1−40_ is cleared more efficiently from the brain in the absence of CD36. Future studies assessing CSF clearance pathways are needed to provide additional support for this conclusion.

## Discussion

We investigated the role of CD36 in BAM in the neurovascular and cognitive dysfunction, and in the underlying parenchymal and vascular amyloid deposition in 15-month-old Tg2576 mice with florid amyloid pathology and cognitive impairment. After establishing and validating a BM chimera-based strategy to target BAM, we found that CD36 deletion from these cells ameliorates neurovascular dysfunction in Tg2576 mice. Loss of CD36 was associated with suppression of ROS production in BAM and reduction of brain Ab_1 − 40_, the Aβ species that accumulates in vessels, but not Ab_1 − 42_, which accumulates in amyloid plaques. Accordingly, neuropathological analysis demonstrated a marked reduction in arteriolar smooth muscle cell damage and CAA, but no reduction in parenchymal amyloid plaques. The rescue of neurovascular function and the reduced oxidative stress and CAA were associated with a profound improvement of cognitive function despite the unchanged parenchymal amyloid load. These findings, collectively, unveil a previously unrecognized role of BAM in the cerebrovascular accumulation of Ab, an effect mediated by CD36, and point to neurovascular dysfunction and CAA as critical pathogenic factors in the attendant cognitive dysfunction.

There is increasing evidence that Aβ clearance depends on cerebrovascular health [2, 6, 13]. Aβ induces major alterations in neurovascular function. In humans with CAA or with APP mutations resulting in increased brain Aβ, as in corresponding animal models, neurovascular reactivity to neural and/or endothelial stimuli is markedly suppressed [16–18, 52, 53, 67–69]. In addition, Aβ alters capillary blood flow distribution, resulting heterogeneous capillary perfusion and reduced efficiency in capillary exchange [70–72]. These deleterious neurovascular effects are in great part mediated by BAM leading to vascular oxidative stress through the CD36-Nox2 ROS-producing signaling pathway [25, 73]. In the present study, we found that CD36 deletion in BAM suppresses Aβ-induced oxidative stress, rescues key features of neurovascular function (functional hyperemia, endothelial and smooth muscle cell vasoreactivity), reduces CAA and improves cognitive function. Therefore, it is reasonable to conclude that the suppression of oxidative stress and improved neurovascular function promotes Aβ vascular clearance reducing its vascular deposition. In support of this hypothesis exogenous Aβ injected into the brain was cleared more efficiently in CD36 null mice, as evidenced by reduced residual Aβ load in the brain and increased blood Aβ in superior sagittal sinus and systemic circulation. While the specific role of BAM CD36 in the mechanisms of Aβ clearance needs to be explored further, the present data provide proof-of-principle evidence that CD36 activation by Aβ promotes vascular retention of Aβ through vascular oxidative stress and neurovascular dysfunction.

Previous studies have raised the possibility that BAM may influence brain Aβ deposition. In a mouse model of mutant APP overexpression (TgCRND8), BAM depletion by icv injection of the toxin chlodronate reduced parenchymal amyloid plaques and increased CAA [74]. In a mouse model of APP overexpression APP (J20) lacking scavenger receptor class-B type I (expressed in all myeloid cells) [75], an increase in both amyloid plaques and CAA in hippocampus was found. Furthermore, recent data suggest that BAM depletion reduces CSF clearance of Aβ and increases parenchymal amyloid load, effects linked to reduced vascular compliance and vasoreactivity [76]. While these observations would advocate for a beneficial role of BAM in preventing amyloid accumulation, our results unveil a previously unrecognized deleterious role of BAM in promoting vascular Aβ accumulation. BAM may well promote CSF clearance, but these cells once engaged by Aβ become a damaging source of vascular oxidative stress through the CD36-Nox2 pathway. Therefore, the neurovascular dysfunction and damage induced by BAM-derived ROS offsets the beneficial effects on CSF clearance.

An important translational implication of this new finding is that targeting CD36 on BAM may preserve the beneficial effects on Aβ clearance while counteracting the deleterious effect of ROS production in vascular structure and function. Therefore, targeting BAM CD36 may be beneficial in CAA or other conditions associated with vascular amyloid accumulation such as the Amyloid-Related Imaging Abnormality (ARIA). ARIA is observed with MRI in cortical areas in up to 47% of patients receiving Aβ immunotherapy at the highest dose [77, 78]. ARIA can underlie vasogenic edema (ARIA-E), or microhemorrhages and cortical superficial siderosis (ARIA-H) [79] and is usually asymptomatic, although in some cases headache, lethargy, confusion, or behavioral changes can occur [78]. ARIA has been attributed to overload of cerebral blood vessels by Aβ peptides released from breakdown of amyloid plaque resulting in vascular dysfunction and damage [78]. Since ARIA is more frequent at high doses of Aβ antibodies, which are also more effective [77, 78], counteracting ARIA may enhance the success of Aβ immunotherapy. Considering the key role of BAM CD36 in Aβ-induced vascular oxidative stress, dysfunction, and amyloid accumulation, blocking this receptor may prove beneficial. Approaches to inhibit CD36 have been developed [80–82] and may provide the opportunity to examine their ability to enhance vascular Aβ clearance.

A potential limitation of our study is related to the use BM chimeras to target BAM. BM transplantation induces severe stress, acute morbidity, and leads to vascular inflammation and BBB opening [83]. However, experiments were performed 3 months after transplantation when these changes are resolved [84]. Accordingly, using this transplantation protocol WT→WT mice have normal neurovascular function, BBB permeability and cognition (present study and refs. [24–26]). Furthermore, WT→Tg2676 chimeras exhibit neurovascular, neuropathological and cognitive alterations identical to those of naïve Tg2576 mice (Present study and ref. [25]), demonstrating that the BM derived BAM repopulating the brain of Tg2576 mice have the same pathogenic effects of native BAM. Therefore, the results of the present study cannot be attributed to confounding effects of BM transplantation.

## Conclusions

We have investigated the role of CD36 in BAM in a mouse model of CAA. First, we established a BM transplantation-based strategy to selectively delete CD36 from BAM in 15-month-old Tg2576 mice with extensive CAA and cognitive impairment. Using this approach, we found that CD36 deletion in BAM suppresses free radical production in these cells and rescues the neurovascular dysfunction induced by Aβ. CD36 deletion in BAM also reduced brain Aβ_1−40_, prevented microvascular smooth muscle cell damage, and ameliorated CAA without affecting parenchyma plaques. Lack of CD36 enhanced the vascular clearance of exogenous Aβ. These beneficial vascular effects resulted in a near complete rescue of cognitive function in 15-month-old Tg2576 mice. Collectively, these data implicate CD36 in BAM in the accumulation of Aβ in cerebral blood vessels and raise the possibility that targeting BAM CD36 is a new therapeutic approach for CAA and other conditions associated with vascular Aβ deposition and damage.

## Supplementary Material

Supplement 1
