# Supplementary material for: Border-associated macrophages promote cerebral amyloid angiopathy and cognitive impairment through vascular oxidative stress
Source: Res Sq. 2023 Apr 28:rs.3.rs-2719812. Preprint. [Version 1] doi: 10.21203/rs.3.rs-2719812/v1 (PMC10168479; doi:10.21203/rs.3.rs-2719812/v1)
Supplement: Supplement 1 [file NIHPPrs2719812v1-supplement-1.pdf]

Supplementary Files

This is a list of supplementary files associated with this preprint. Click to download.

- floatimage1.jpeg
- AdditionalFiles.docx
